# Going beyond the coronaries: Routine cardiovascular risk assessment reveals rare incidental thymoma

**DOI:** 10.1016/j.radcr.2023.01.020

**Published:** 2023-02-03

**Authors:** Daniel Karlsberg, Henry Steyer, Nicole Grignoli, John Rumberger

**Affiliations:** a Cardiovascular Research Foundation of Southern California, Los Angeles, CA, USA; b NYU Langone Health, New York, USA; c Princeton Longevity Center, Princeton, New York, USA; d Keck School of Medicine of USC, Los Angeles, CA, USA

**Keywords:** Incidental mass, Thymoma, Mediastinal mass, Cardiac computed tomographic angiography, Pericardial mass, coronary artery calcium score

## Abstract

Thymomas are rare anterior mediastinal masses that present with local or paraneoplastic symptoms. Definitive diagnosis requires tissue sampling but early detection leads to early intervention and improved outcomes. We present a case where routine cardiovascular risk assessment identified an incidental and rare thymoma. Final specimen pathology revealed a Thymoma WHO Type AB (30% A, 70% B). Routine cardiovascular risk assessment which often includes coronary artery calcium scanning and cardiovascular computed tomographic angiography may reveal pathology beyond the coronary arteries. Early detection of asymptomatic mediastinal masses facilitates early intervention and can improve outcomes.

## Introduction

Thymomas are rare anterior mediastinal masses that present with local or paraneoplastic symptoms. Definitive diagnosis requires tissue sampling but early detection leads to early intervention and improved outcomes. We present a case where routine cardiovascular risk assessment identified an incidental and rare thymoma.

## Case presentation

**Fig. 1:**
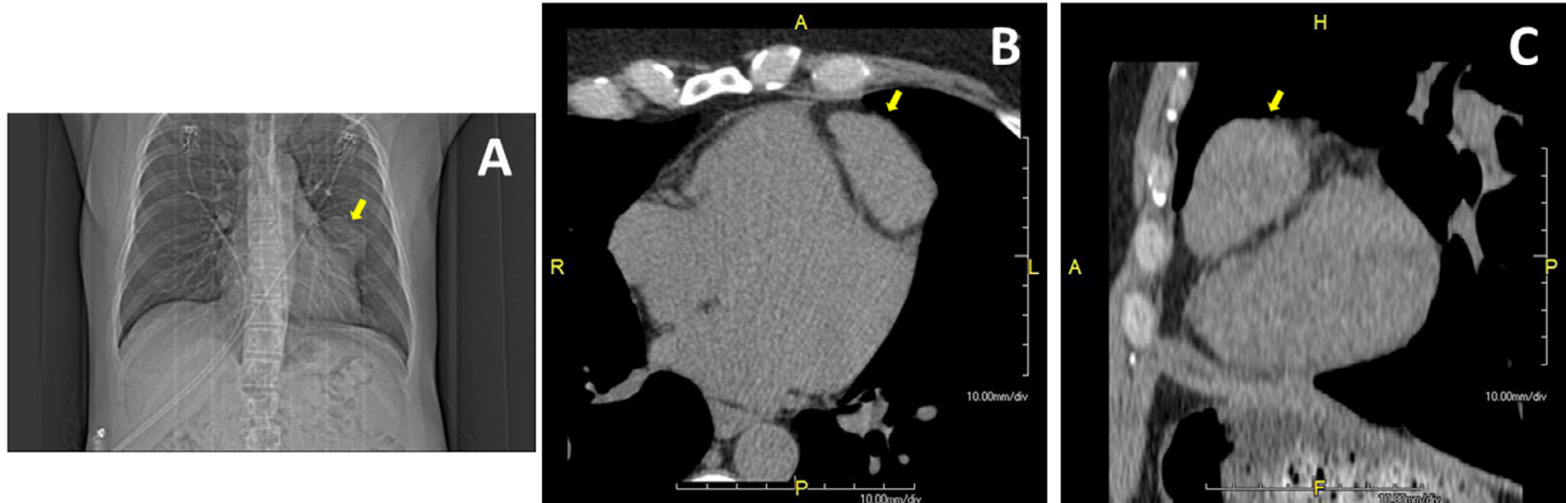
(A-C) Coronary artery calcium scan, as well as scout images, revealed a coronary calcium score of zero and a 5.3 × 3.8 cm anterior mediastinal mass (*yellow arrows*).

**Fig. 2:**
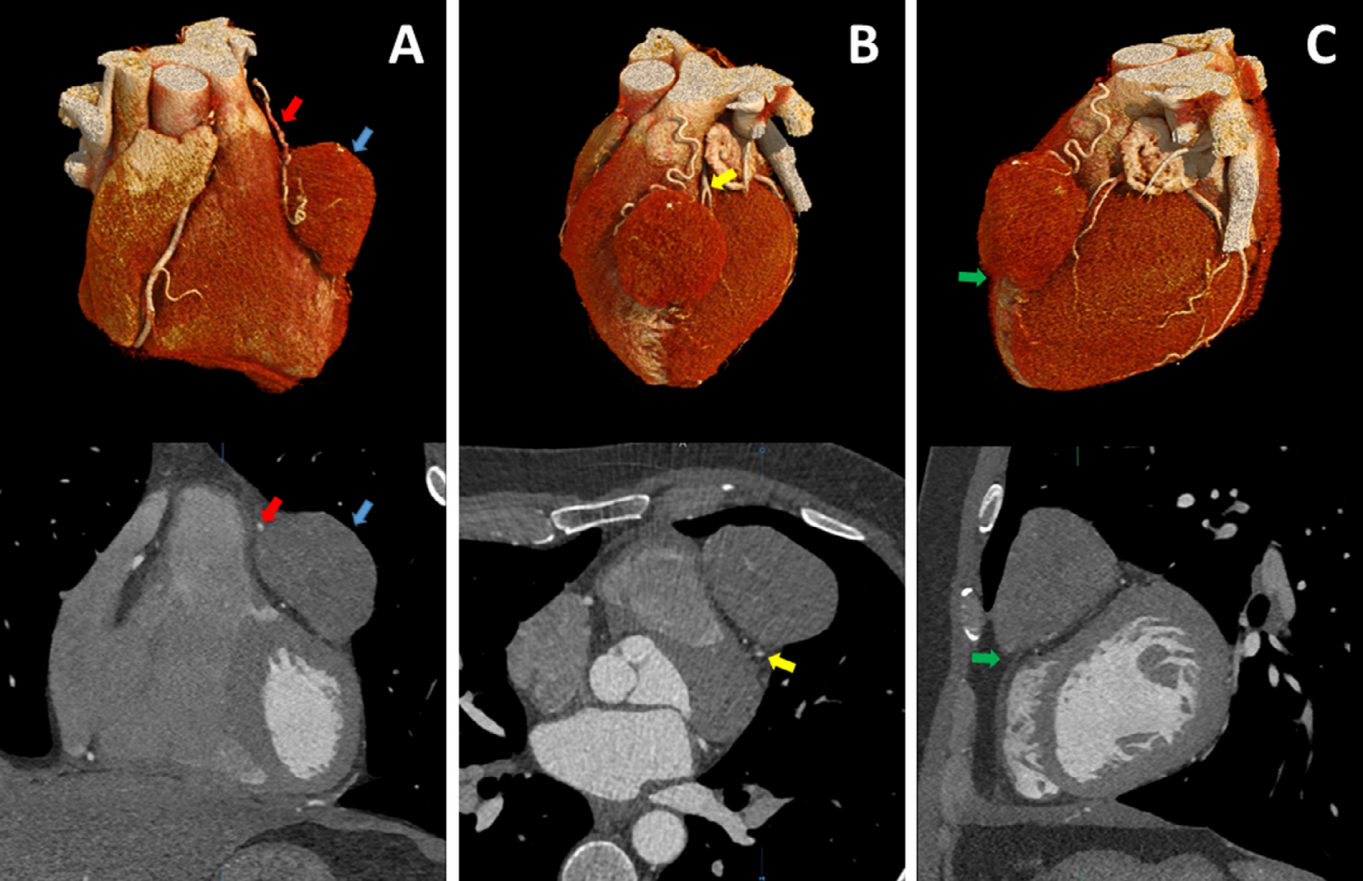
Cardiovascular computed tomographic angiography demonstrating mass borders (A, *blue arrows*), independent mass blood supply (A, *red arrows*), mass proximity to the left anterior descending coronary artery (B, *yellow arrows*), and left and right ventricles (C, *green arrows*).

A 48-year-old male presented for cardiovascular risk assessment. History was consistent with an intermediate cardiovascular risk profile while physical exam, laboratory evaluation, and electrocardiogram were unremarkable. Coronary artery calcium scanning, as well as scout images, revealed a coronary calcium score of zero and a 5.3 × 3.8 cm anterior mediastinal mass (Figs. 1A-C, *yellow arrows*). Further assessment with cardiovascular computed tomographic angiography (CCTA) was performed given mass size and proximity to anterior cardiac structures. CCTA clearly defined mass borders (Fig. 2A, *blue arrows*), independent mass blood supply (Fig. 2A, *red arrows*), mass proximity to the left anterior descending coronary artery (Fig. 2B, *yellow arrows*), and left and right ventricles (Fig. 2C, *green arrows*).

Differential diagnosis included pericardial cyst, benign tumor such as hemangioma, or malignancy such as thymoma, thymic carcinoma, teratoma, lymphoma, angiosarcoma, germ cell cancer, or peripheral lung cancer. Cardiac MRI was performed to evaluate for mass invasion which demonstrated a well circumscribed pericardiac mass but no invasion.

The patient was referred for robot-assisted resection. Final specimen pathology revealed a Thymoma WHO Type AB (30% A, 70% B). Radical thymectomy and radiation therapy were proposed but ultimately active surveillance with periodic CCTA was pursued. Follow-up imaging at 1 year demonstrated no recurrence.

## Discussion

Thymomas account for 20% of anterior mediastinal masses yet have an incidence of 0.13 per 100,000 person-years [1]. Presentation occurs most commonly between 40 and 60 years old. Clinical presentations range from asymptomatic to severe thoracic symptoms; chest pain, dyspnea, and phrenic nerve palsy or paraneoplastic syndromes such as myasthenia gravis and blood cell dyscrasia. Staging systems include the Masaoka and tumor, nodes, metastasis (TNM) systems which associate invasion and spread with poor outcomes. In this case, CCTA suggested an easily resectable, well-circumscribed mass with no high-risk features or metastasis. Final pathology revealed nonaggressive AB thymoma. Therefore, active surveillance with periodic imaging was recommended [2].

## Conclusion

Routine cardiovascular risk assessment which often includes coronary artery calcium scanning and CCTA may reveal pathology beyond the coronary arteries. Early detection of asymptomatic mediastinal masses facilitates early intervention and can improve outcomes. Cardiac imagers must be familiar with rare incidental findings and their management.

## Patient consent

The patient in this case report has formally consented to publication of clinical context and images.
